# International Homœopathic Congress

**Published:** 1896-03

**Authors:** W. A. Dewey

**Affiliations:** Secretary, 170 West Fifty-fourth Street, New York


### INTERNATIONAL HOMŒOPATHIC CONGRESS.

The Committee on International Congress desires to announce that after nearly four months' unceasing effort, it has been totally unable to secure special rates or accommodations for the Congress in London. The date of the Congress having been set at a time when the steamships are overcrowded, the companies, not wishing the business, refuse to consider it.

The White Star Line agreed, however, to reserve a very limited number of berths on the "Britannic," sailing hence on June 24th, and the Cunard Line has also agreed to reserve a few berths on their steamer "Umbria," sailing on June 27th. These are special concessions. The berths in each case will be held *only* until March 15th. The prices range from $108 to

$150 each, for four persons in a room, for the round-trip. Reservations can only be made by a deposit of $25. Those desiring to avail themselves of these sailings should apply at *once* to the Secretary.

W. A. DEWEY, M. D.,
*Secretary*,
170 West Fifty-fourth Street, New York.

